# Time-dependent pharmacodynamics of amikacin on *Mycobacterium abscessus* growth and resistance emergence

**DOI:** 10.1128/spectrum.03222-23

**Published:** 2024-01-18

**Authors:** Joy E. Gibson, Nishant Nandanwar, Michael N. Neely

**Affiliations:** 1 Division of Infectious Diseases and the Laboratory of Applied Pharmacokinetics and Bioinformatics, The Saban Research Institute, Children’s Hospital Los Angeles, Los Angeles, California, USA; 2 Department of Pediatrics, University of Southern California Keck School of Medicine, Los Angeles, California, USA; University of Nebraska Medical Center, Omaha, Nebraska, USA

**Keywords:** amikacin, aminoglycosides, *Mycobacterium abscessus*, NTM, pharmacokinetics, pharmacodynamics, hollow fiber

## Abstract

*Mycobacterium abscessus* pulmonary disease is increasing in prevalence globally, particularly for individuals with cystic fibrosis. These infections are challenging to treat due to a high rate of resistance. Amikacin is critical to treatment, but the development of toxicity, amikacin resistance, and treatment failure are significant challenges. Amikacin has been characterized previously as peak-dependent and extended-interval dosing is commonly used. In our hollow fiber infection model of *M. abscessus*, amikacin exhibited time-dependent rather than the expected peak-dependent pharmacodynamics. Humanized amikacin exposures with more frequent, short-interval dosing (continuous infusion or every 12 hours) yielded improved microbiological response compared to extended-interval dosing (every 24 hours or 1–3 times per week). Short-interval dosing inhibited growth with a mean (SD) maximum Δlog_10_ colony forming units of −4.06 (0.52), significantly more than extended-interval dosing (*P* = 0.0013) every 24 hours, −2.40 (0.58), or 1–3 times per week, −2.39 (0.38). Growth recovery, an indicator of resistance emergence, occurred at 6.56 (0.70) days with short-interval dosing but was significantly earlier with extended-interval dosing (*P* = 0.0032) every 24 hours, 3.88 (0.85) days, and 1–3 times per week, 3.27 (1.72) days. Microbiological response correlated best with the pharmacodynamic index of %T > minimum inhibitory concentration (MIC), with an EC_80_ for growth inhibition of ~40%T > MIC. We used a previously published population model of amikacin to determine the probability of achieving 40%T > MIC and show that current dosing strategies are far below this target, which may partially explain why treatment failure remains so high for these infections. These data support a cautious approach to infrequent amikacin dosing for the treatment of *M. abscessus*.

**IMPORTANCE:**

Pulmonary disease caused by *Mycobacterium abscessus* complex (MABSC) is increasing worldwide, particularly in patients with cystic fibrosis. MABSC is challenging to treat due to high levels of antibiotic resistance. Treatment requires 2–4 antibiotics over more than 12 months and has a significant risk of toxicity but still fails to eradicate infection in over 50% of patients with cystic fibrosis. Antibiotic dosing strategies have been largely informed by common bacteria such as *Pseudomonas aeruginosa*. The “pharmacodynamic” effects of amikacin, a backbone of MABSC treatment, were thought to be related to maximum “peak” drug concentration, leading to daily or three times weekly dosing. However, we found that amikacin MABSC kill and growth recovery, an indicator of antibiotic resistance, are dependent on how long amikacin concentrations are above the minimum inhibitory concentration, not how high the peak concentration is. Therefore, we recommend a re-evaluation of amikacin dosing to determine if increased frequency can improve efficacy.

## INTRODUCTION

Non-tuberculous mycobacteria (NTM) disease has been increasing in frequency globally (1–4). Estimates of disease burden vary widely, but one recent study in the United States demonstrated an increase from 6.8 to 11.7 per 100,000 persons annually between 2008 and 2015 (4). Patients with cystic fibrosis (CF) are among the most vulnerable. In 2020 alone, 10% of individuals with CF had NTM isolated from sputum (5). *Mycobacterium abscessus* complex (MABSC) is a group of rapidly growing NTM that cause a significant proportion of disease, accounting for 36% of NTM-positive cultures in CF patients in 2020 (5). MABSC is particularly challenging to treat due to a high rate of intrinsic and acquired antibiotic resistance (6). The treatment of pulmonary MABSC disease requires multiple antibiotics for over 12 months and has a high rate of toxicity (7, 8). Despite this, treatment failure still occurs in over 50% of patients (9, 10). More studies regarding optimal treatment are needed to improve patient outcomes.

A backbone of MABSC treatment is the aminoglycoside amikacin. Recommended dosing of amikacin is by intravenous administration for a minimum of 3 months, and this is often followed by inhalation for the remainder of therapy (7, 8). Aminoglycosides are described as having peak-dependent pharmacokinetics-pharmacodynamics (PK-PD) against Gram-negative bacteria, allowing them to be administered with longer dosing intervals (11–13). Therefore, in an effort to reduce toxicity from prolonged treatment with amikacin for MABSC, less frequent dosing, particularly thrice weekly, is advocated (7, 8). However, the PK-PD studies to support this against MABSC are limited.

Ferro et al. (14) used an *in vitro* hollow fiber infection model (HFIM) to characterize amikacin PK-PD. They found that the amikacin effect correlated best with the Peak:minimum inhibitory concentration (MIC) ratio. However, there did appear to be some correlation with 24-hour area under the curve (AUC_24_) and the effect depended on the duration of the experiment, with some timepoints showing a better correlation with %T > MIC. In our current study, we used a similar HFIM to quantify bacterial kill and development of resistance of *M. abscessus* subsp. *abscessus* (Mab) with amikacin treatment to further define PK-PD of amikacin against MABSC. This work expands on the study by Ferro et al. to include a wider range of dosing frequencies, from continuous infusion to weekly, and the additional analysis of pediatric PK parameters to relate to clinical practice.

## RESULTS

**Fig 1:**
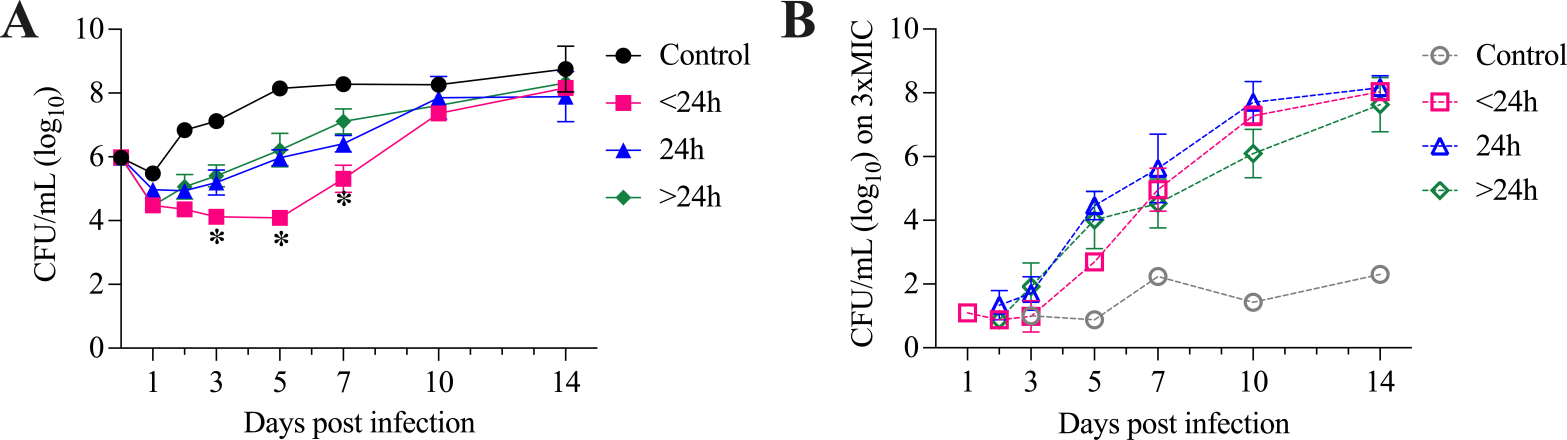
Growth of Mab in the HFIM with amikacin treatment. CFU on MH2 agar plates without (closed symbols, **A**) or with 3xMIC amikacin (open symbols/dashed lines, **B**) over 14 days. Amikacin was administered at different concentrations and dosing frequencies to approximate human pediatric doses with an infusion time of 30–60 min and half-life of 3 hours. Control arms did not receive antibiotic or vehicle. Amikacin dosing frequencies are summarized as <24 hours (pink; continuous infusion, *n* = 1, or every 12 hours, *n* = 4), every 24 hours (blue; *n* = 4), or >24 hours (green; thrice weekly, *n* = 2, or every 7 days, *n* = 1). Mean ± SEM of CFU/mL grouped by dosing frequency is shown. On days 3, 5, and 7, CFU/mL was significantly lower for amikacin dosed <24 hours than for all other conditions, *P < 0.05. During the early time points, resistant isolates were below the limit of detection in the quantity of bacteria plated on 3xMIC amikacin plates. Data shown in B are only reported when resistant isolates were above the threshold to allow quantification.

**Fig 2:**
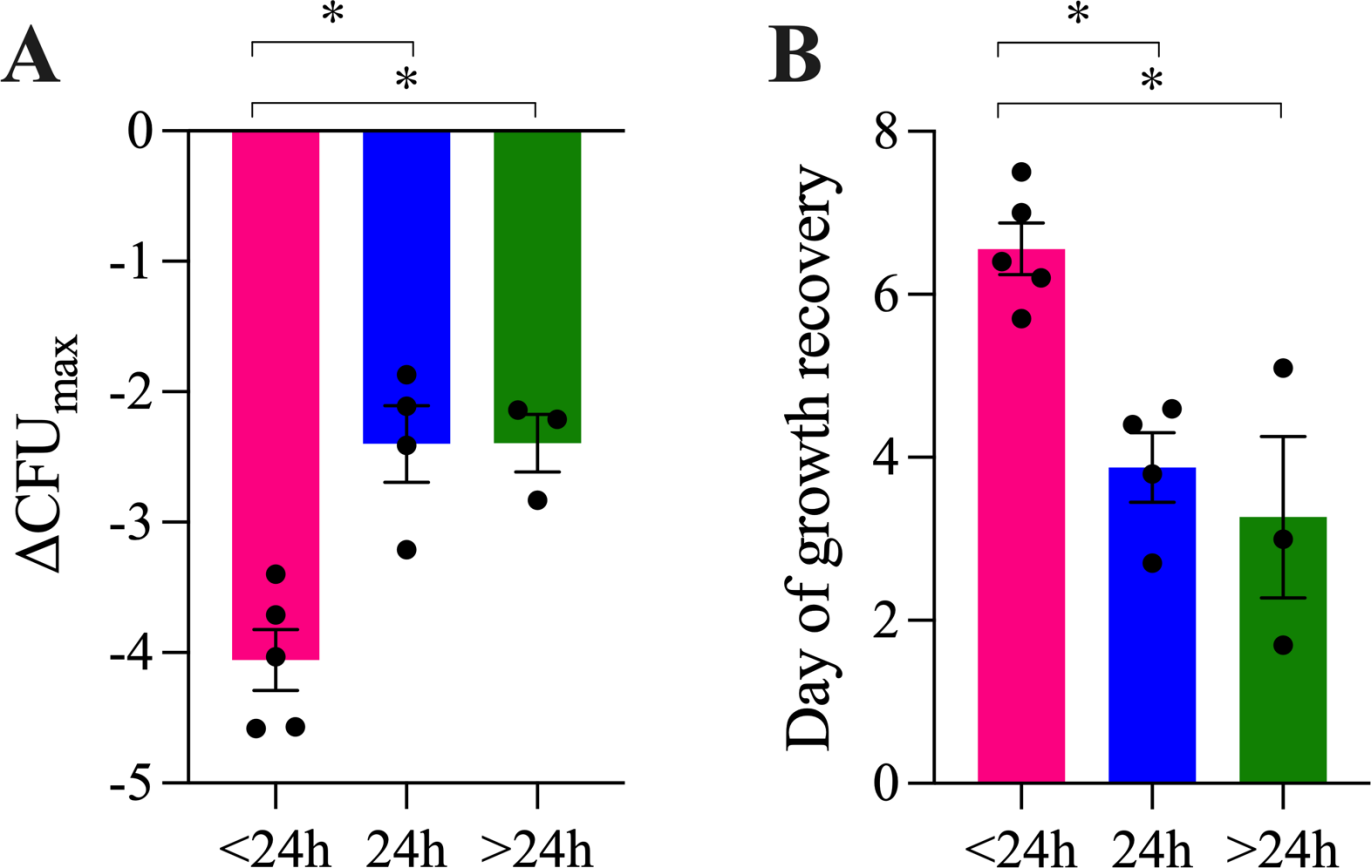
Microbiological response of Mab in the HFIM with amikacin treatment. Data were analyzed for results grouped by amikacin dosing frequencies of <24 hours (continuous infusion, *n* = 1; or every 12 hours, *n* = 4), every 24 hours (*n* = 4), or >24 hours (thrice weekly, *n* = 2; or every 7 days, *n* = 1). Mean ± SEM of ΔCFU_max_ (**A**), defined as the maximum decrease in CFU compared to control samples obtained at the same timepoint, and day of growth recovery (**B**), defined as the day that CFU had rebounded by 1 log10 above CFU_min_ in a treatment arm, linearly interpolated from the surroundings days when CFU samples were collected. For both indicators of microbiological response, the effect was significantly greater with amikacin dosed <24 hours compared to the other conditions, *P < 0.05.

We quantified bacterial growth in the HFIM over 14 days with various amikacin dosing conditions (Table 1; Fig. 1; Fig. S1). The growth curves when amikacin was administered with more frequent, short-interval dosing (continuous infusion or every 12 hours) showed greater growth inhibition relative to extended-interval dosing from days 3 to 7 (*P* < 0.0001, analysis of variance [ANOVA], as shown in Fig. 1D). Maximal growth inhibition occurred when amikacin was administered continuously (ΔCFU_max_ −4.58 log_10_ CFU) or every 12 hours (ΔCFU_max_ −3.4 to −4.57 log_10_ CFU). Growth inhibition was decreased when amikacin was given as extended-interval dosing, including every 24 hours (ΔCFU_max_ −1.87 to −3.21 log_10_ CFU), thrice weekly (ΔCFU_max_ −2.14 to −2.83 log_10_ CFU), and every 7 days (ΔCFU_max_ −2.21 log_10_ CFU). These data are reported numerically in Table 1. Overall, ΔCFU_max_ was significantly greater when amikacin was dosed continuously or every 12 hours, with a mean (SD) of −4.06 (0.52) log_10_ CFU, compared to dosing every 24 hours, with −2.40 (0.58) log_10_ CFU, or dosing 1–3 times per week, with −2.39 (0.38) log_10_ CFU (*P* = 0.0013, ANOVA), as shown in Fig. 2A.

**TABLE 1:** Summary of HFIM dosing regimens, pharmacodynamic indices, and microbiological effect^*a*^

Target C_max_ (μg/mL)^*b*^	Infusion time (min)	1 hour C_max_ (μg/mL)	Frequency	Approximate human daily dose^*c*^	%Time > MIC^*d*^	Peak:MIC^*d*^	AUC_24_:MIC^*d*^	ΔCFU^*e*^_max_	Day of growth recovery*^f^*
15	Continuous	15.0	Continuous	20 mg/kg	100.0	1.9	45.0	−4.58	6.2
20.3	30	18.1	q12h	10 mg/kg	36.2	2.3	21.8	−3.71	5.7
22.3	30	19.9	q12h	12 mg/kg	40.0	2.5	24.0	−4.03	7.5
23.3	60	23.3	q12h	15 mg/kg	44.7	2.9	26.5	−4.57	7.0
50.5	30	45.0	q12h	25 mg/kg	70.0	5.6	54.0	−3.40	6.4
12.6	30	11.2	q24h	3.5 mg/kg	9.0	1.4	7.2	−1.87	4.4
40.1	30	35.7	q24h	10 mg/kg	30.8	4.5	22.9	−2.11	2.7
45.7	30	40.7	q24h	12 mg/kg	33.2	5.1	26.1	−2.41	3.8
52.5	60	52.5	q24h	15 mg/kg	37.5	6.6	31.7	−3.21	4.6
71.2	30	63.4	TIW^*g*^	20 mg/kg	17.7	7.9	18.1	−2.14	1.7
149.6	30	133.3	TIW	40 mg/kg	23.5	16.7	38.1	−2.83	5.1
90	30	80.2	q7 days	25 mg/kg	6.5	10.0	7.4	−2.21	3.0

^*a*^ Notes: HFIM conditions modeled a half-life of 3 hours in all experiments.

^*b*^ Target C_max_ is defined as the free amikacin concentration at the end of amikacin infusion.

^*c*^ Approximate human daily dose that corresponds with the targeted 1 hour C_max_ based on our Pmetrics model using pediatric dosing.

^*d*^ For all indices, an MIC value of 8 μg/mL was used.

^*e*^ ΔCFU_max_ is the maximum decrease in CFU compared to control samples obtained at the same time.

^*f*^ Day of growth recovery is the day that CFU had rebounded by 1 log_10_ above CFU_min_ in a treatment arm, linearly interpolated from the surroundings days when CFU samples were collected.

^*g*^ TIW, three times weekly.

We also quantified the emergence of amikacin resistance by growth on agar containing amikacin at 3× MIC (Fig. 1B). For the ATCC strain of Mab used, amikacin MIC was 8 µg/mL. At baseline, mean (SD) amikacin phenotypic resistance frequency in this Mab strain is 1.9 (2.4) × 10^−7^ (*n* = 3). We first detected resistant colonies between 1 and 7 days, with resistance emergence varying between dosing conditions. Resistance did not increase over time in control arms. However, with amikacin monotherapy, regardless of dosing frequency or concentration, resistance rapidly emerged, and the resistant organisms fully replaced the susceptible bacterial population within 7–14 days. To control for different times to first resistant CFU detection and better compare regimens, we defined our resistance endpoint of growth recovery as 1 log_10_ CFU increase above the minimum CFU. More frequently, short-interval dosing of amikacin delayed growth recovery until 5.7–7.5 days with amikacin continuous or every 12-hour dosing, compared to extended-interval dosing with 2.7–4.6 days with amikacin every 24 hours, and 1.7–5.7 days with amikacin thrice weekly or once weekly (Table 1). Overall, growth recovery was significantly delayed when amikacin was given with short-interval dosing, continuously or every 12 hours, occurring at a mean (SD) of 6.56 (0.70) days post infection, compared to extended-interval dosing every 24 hours with growth recovery at 3.88 (0.85) days, or dosing 1–3 times per week with growth recovery at 3.27 (1.72) days (*P* = 0.0032, ANOVA), as shown in Fig. 2B.

**Fig 3:**
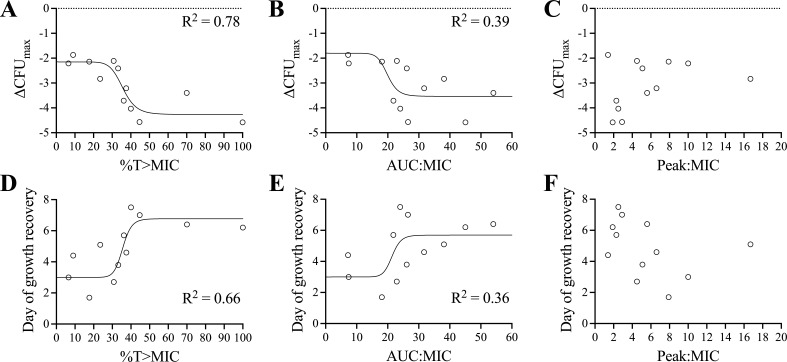
Sigmoidal inhibitory Emax regression models of PK-PD indices describing the microbiological response of Mab to amikacin in the HFIM. ΔCFU_max_ (**A–C**) and day of growth recovery (**D–F**) are shown relative to %T > MIC (**A and D**), AUC_24_:MIC (**B and E**), and Peak:MIC (**C and F**). *R*^2^ associated with each curve is shown. Peak:MIC did not fit any sigmoidal regression model.

Based on our non-linear regression analysis of ΔCFU_max_ or day of growth recovery (DGR), which compared %T > MIC, AUC_24_:MIC, or Peak:MIC, the best fit occurred with %T > MIC, with an *R*^2^ of 0.78 for ΔCFU_max_, an EC_50_ of 36%T > MIC, and Akaike information criterion (AIC) of 24.8 (Fig. 3). For day of growth recovery, the regression had an *R*^2^ of 0.66, an EC_50_ of 36%T > MIC, and AIC of 44.1. AUC_24_:MIC was a worse predictor of ΔCFU_max_ than %T > MIC, with *R*^2^ of 0.39 and AIC of 36.5. For day of growth recovery, AUC_24_:MIC was also worse, *R*^2^ was 0.36, and AIC was 51.9. Peak:MIC did not fit any sigmoidal regression curve. Based on the highest *R*^2^ and the lowest AIC for both ΔCFU_max_ and day of growth recovery, %T > MIC was the best predictor. This was consistent at every timepoint when analyzed independently for microbial kill based on the AIC score (Table S1).

**Fig 4:**
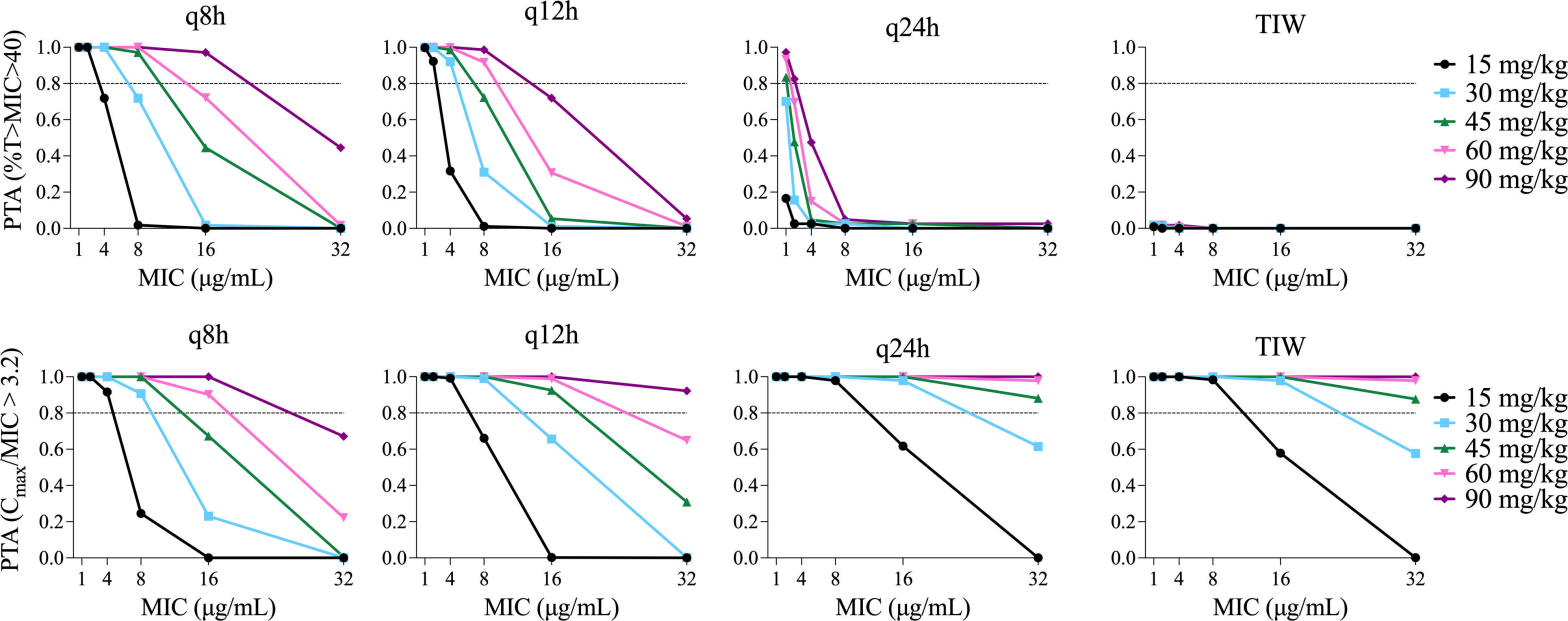
PTA. For each plot, PTA was determined from 1,000 simulated concentration-time profiles at total daily dosages of 15, 30, 45, 60, and 90 mg/kg/day divided every 8, 12, or 24 hours (q8h, q12h, and q24h) and also given TIW. Top row target was %T > MIC ≥ 40%, which was the EC_80_ based on our sigmoidal regression analysis for ΔCFU_max_. In the bottom row, the target was Peak:MIC ≥ 3.2 as suggested by Ferro et al. (14). In all plots, the goal PTA was >0.8 as indicated by the horizontal dotted lines.

The probability of target attainment (PTA) analysis for expected concentration-time profiles arising from pediatric dosing regimens is shown in Fig. 4. Cognizant of the increased risk of nephrotoxicity with high trough concentrations of aminoglycosides, based on our regression model, we chose %T > MIC of 40% as a reasonable target which would achieve ~80% of maximal growth inhibition (EC_80_) without unnecessarily prolonging high concentrations. Our goal PTA was 80% of simulated concentration-time profiles achieving this target for a given dosage regimen. Clinical isolates of Mab have higher MICs on average than typical Gram-negative pathogens. Ferro et al. reported a mean MIC for Mab of 16 µg/mL among 44 clinical isolates (14). All dosing regimens failed to achieve 80% PTA for an MIC of 16 µg/mL except for 90 mg/kg (total body weight) per day divided every 8 hours, which reached 97% PTA. For an MIC of 8 µg/mL, amikacin doses of 45–90 mg/kg/day divided every 8 hours and 60–90 mg/kg/day divided every 12 hours achieved 80% PTA. Amikacin dosed every 24 hours only achieved >80% PTA for MIC ≤2 µg/mL if the dose was 90 mg/kg and for MIC ≤1 µg/mL if the dose was 60 mg/kg. Thrice weekly dosing up to 90 mg/kg/dose did not meet 80% PTA for any MIC. None of the current recommended clinical dosing strategies [10–30 mg/kg/dose daily or thrice weekly (7, 8)] reached >80% PTA, even with very low MICs.

Amikacin has been described to follow peak-dependent PD for other organisms such as *Pseudomonas*, and a target Peak:MIC ratio of 3.2 was proposed by Ferro et al. for Mab (14). In our model, this Peak:MIC target was more readily achieved than our proposed %T > MIC targets, particularly with every 24-hour or thrice-weekly dosing (Fig. 4). When amikacin was dosed every 24 hours or thrice weekly, 45–90 mg/kg/day yielded >80% PTA for an MIC of 16 µg/mL. However, Peak:MIC was not identified as the PK-PD link in our hollow fiber system.

**Fig 5:**
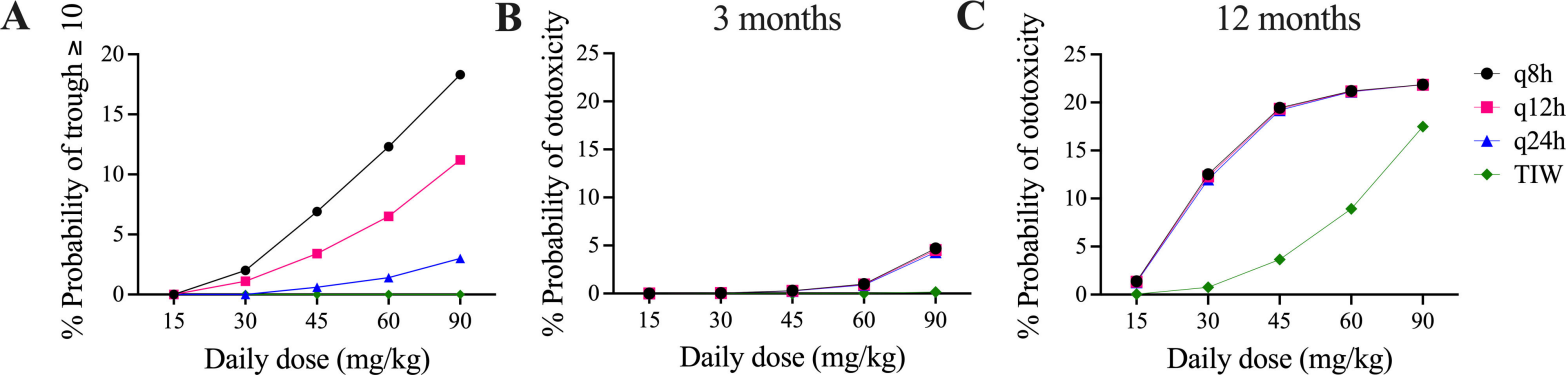
Probability of nephro- and ototoxicity by regimen. Probability of toxicity was determined from 1,000 simulated concentration-time profiles at total daily dosages of 15, 30, 45, 60, and 90 mg/kg/day divided every 8, 12, or 24 hours (q8h, q12h, and q24h) and also given TIW. Nephrotoxicity threshold was defined as an amikacin trough of ≥10 µg/mL (**A**), and probability of ototoxicity for given cumulative AUC over 3 months (**B**) and 12 months (**C**) was according to the function in Modongo et al. (16).

Another important consideration for amikacin dosing is to minimize the risk of nephro- and ototoxicity. Nephrotoxicity has been associated with higher trough concentrations, with a proposed trough cutoff of 10 µg/mL (15). Our PTA for a trough concentration of ≥10 µg/mL (with a goal to minimize attainment in this case) was not surprisingly highest with 90 mg/kg/day divided every 8 hours, which resulted in 18% PTA (Fig. 5A). However, typical clinical dosing regimens of 15–30 mg/kg/day had a low probability of achieving ≥10 µg/mL, with the highest PTA being 2% for 30 mg/kg/day divided every 8 hours. The risk of ototoxicity has been associated with cumulative AUC, rather than trough, and Modongo et al. proposed a threshold of 87,232 mg/L*hours for ototoxicity, which they associated with a 10% risk of ototoxicity (16). In our model, with 1 year of therapy, dosing regimens of 45–90 mg/kg/day resulted in 19%–21% PTA for ototoxicity for all regimens except thrice weekly (Fig. 5C). However, the ototoxicity risk was substantially lower for the typical duration of intravenous amikacin therapy of 3 months, with 90 mg/kg/day reaching only 5% and all other regimens resulting in less than 1% PTA for ototoxicity (Fig. 5B).

## DISCUSSION

As attractive as less frequent aminoglycoside dosing is to both simplify regimens and reduce toxicity, published evidence to support less frequent dosing of aminoglycosides, especially thrice weekly, does not adequately address efficacy against MABSC in terms of either kill magnitude or suppression of resistance emergence. While a recent Cochrane review showed equivalent short-term efficacy and reduced toxicity with q24h aminoglycoside treatment relative to three times daily dosing (17), the indication was for *Pseudomonas aeruginosa* treatment in CF, not MABSC, the review did not assess long-term efficacy or the emergence of resistance, and TIW dosing was not included. There are no comprehensive clinical studies comparing amikacin dosing frequencies for MABSC pulmonary disease. Our data suggest that the clinical response of Mab to amikacin may be different than for Gram-negative rods like *Pseudomonas*.

Aminoglycosides are established as peak-dependent drugs based on pre-clinical and clinical studies of Gram-negative bacteria, which grow faster and typically have lower aminoglycoside MICs than MABSC (18, 19). Our data show that growth inhibition, as indicated by maximum change in CFU, is greatest with short-interval dosing (less than 24 hours). Short-interval dosing also provides more favorable outcomes regarding the emergence of resistance, as indicated by the day of growth recovery. This suggests more time-dependent PK-PD for the effect of amikacin on Mab. Non-linear regression analysis supports this conclusion, with the strongest correlation to effect occurring with %T > MIC. In contrast to what has been shown for other organisms, there was no correlation of effect with Peak:MIC in our studies. Ferro et al. discuss a PK-PD “wobble” and report that at some times in their hollow fiber system, amikacin behaved with time-dependent PD against Mab as we found. We also analyzed the PK-PD relationship at each time point but consistently found time dependence as the superior predictor of either kill or suppression of resistance.

The reasons for these unexpected findings are not clear. As a *Mycobacterium*, the kinetics of growth for MABSC are very different from typical gram-negative pathogens, which is likely to impact the duration of the effect of antibiotics. Peak-dependent antibiotics typically have a prolonged post-antibiotic effect (PAE), which allows for continued growth inhibition even during times when the concentration is below the MIC. Studies have shown a prolonged PAE for aminoglycosides against Gram-negative pathogens (20), but this has not been studied for MABSC. With the slow rate of growth of MABSC, it is possible that any PAE that occurs is not adequate to maintain growth suppression. Furthermore, the amikacin MIC for MABSC is generally much higher than for Gram-negative pathogens. The optimal Peak:MIC is suggested to be ≥8 for Gram-negative rods (19), but this goal is not achieved for MICs of ≥8 µg/mL, which are typical for MABSC. Work by Ferro et al. suggests that this goal may be lower for MABSC but can still be difficult to achieve (14). It is reasonable to conclude that peak-dependence may be less relevant clinically in organisms with higher amikacin MICs, such as MABSC, leading to a more important role for time and AUC.

Our findings indicate a need to re-evaluate amikacin dosing recommendations for MABSC pulmonary disease. To determine what the optimal dosing strategy may be, we used computer modeling and simulation to assess the PTA to achieve growth inhibition and to avoid nephro- and ototoxicity. When using a target of 40%T > MIC for clinical effect, more frequent dosing is best. However, this does also increase the risk of nephrotoxicity, particularly when dosing every 8 hours. Therefore, a balance between these factors is needed. When the same total daily dose is used, the risk of ototoxicity is equivalent for all dosing frequencies, except for thrice weekly. However, thrice weekly dosing results in extremely poor %T > MIC PTA, and our data suggest that this strategy may compromise efficacy. Current CF Foundation and American Thoracic Society guidelines for treatment of MABSC pulmonary disease recommend 10–30 mg/kg/dose daily or thrice weekly (7, 8). Our data show that the currently recommended amikacin dosing would be likely to achieve an adequate Peak:MIC target but would not reach our proposed %T > MIC targets. This may, in part, explain why clinical failure of MABSC treatment is so high despite what is thought to be adequate dosing with high target attainment when the target is peak concentration. The reported clinical success rates of less than 50% (9, 10) are more in line with the lower probabilities of target attainment for time above MIC that we found.

As discussed above, less frequent dosing has long been favored to reduce toxicity risk. However, our data indicate that a higher dose of 30–45 mg/kg/day divided every 8 to 12 hours would likely improve efficacy while still maintaining a nephrotoxicity risk below 10% and an acceptable ototoxicity risk of <1% for a 3-month intravenous amikacin treatment regimen. This toxicity risk is a modeled prediction and would need to be closely monitored if more frequent dosing is used clinically.

It is important to note that growth inhibition and the emergence of resistance were suboptimal for all dosing strategies with amikacin monotherapy, even continuous infusion. Current guidelines include treatment with 3–4 antibiotics, and our findings emphasize the importance of such combination therapy. We examined amikacin monotherapy in only one laboratory strain of Mab, and it will be important to confirm these findings in clinical strains and in combination therapy experiments before generalizing to clinical dosing strategies. While we intend to study PK-PD of combination therapy in the HFIM, it was important to start with amikacin monotherapy for several reasons. First, ascertaining the optimal PK-PD relationship of multiple drugs simultaneously in the HFIM is factorially expensive and complex, necessitating knowledge of optimal targets of individual agents to reduce the number of combinations. Second, amikacin is often clinically combined with oral drugs, and if adherence or absorption of these drugs is compromised, there may be disproportionate reliance upon amikacin, making knowledge of mono-therapeutic PK-PD more important. Third, some isolates may be only susceptible to amikacin, and although combination therapy is still indicated due to uncertainty about the predictive strength of laboratory resistance on clinical outcomes, amikacin again may be the most active agent in the regimen.

In conclusion, we found that at exposures associated with current CF dosing regimens and typical pediatric PK, amikacin exerts time-dependent PD against Mab with respect to kill and resistance emergence, which may be a function of dosing intervals that exceed the PAE after a given peak concentration. Further investigation of the amikacin PAE and these findings is warranted. Ongoing and future clinical studies of Mab treatment should incorporate PK-PD analysis correlated with clinical response to verify the optimal dosing regimen(s). Our findings indicate that higher dosing of 30–45 mg/kg/day divided every 8–12 hours, particularly in the initial intensive phase of treatment, may be warranted to improve efficacy.

## MATERIALS AND METHODS

### Bacteria and reagents

For all experiments, *M. abscessus* ATCC 19977 (American Type Culture Collection) was used. Stock cultures were stored at −80°C in Middlebrook 7H9 broth (BD Difco) supplemented with 10% oleic acid albumin dextrose catalase (BD Biosciences) and 20% glycerol. With each experiment, a fresh vial of bacteria was thawed, centrifuged at 8,000 rpm for 5 min, and the bacterial pellet was resuspended in fresh media. Bacteria were incubated at 37°C for ~24 hours until at logarithmic growth phase prior to diluting for initial inoculum. With the exception of glycerol stocks, all bacterial culture was carried out in Cation-adjusted Mueller-Hinton II broth (MH2; Sigma-Aldrich or VWR). Amikacin sulfate powder was purchased from Sigma-Aldrich. Stock amikacin was stored at 25 mg/mL in sterile ddH2O at −20°C.

### MIC and mutation frequency

We determined the MIC for amikacin using the standard broth microdilution method established by CLSI (21). We quantified the baseline frequency of amikacin resistance by diluting bacteria to ~1 × 10^8^ CFU/mL and plating a total of 3 × 10^8^ CFU onto MH2 agar containing amikacin at 3xMIC (24 µg/mL), distributing 200 µL of bacteria per 10 cm agar dish, and incubating at 37°C for 4–5 days. We also quantified total CFU/mL by serial dilution and plating onto antibiotic-free plates. Amikacin resistance mutation frequency was defined as the number of CFU on amikacin plates relative to the total CFU plated.

### Hollow fiber infection model

In a polysulfone cartridge (C2011, FiberCell Systems), we treated Mab with amikacin sulfate (Sigma-Aldrich) per established protocol (22–25). Briefly, bacteria were inoculated at a starting inoculum of 1 × 10^6^ CFU/mL into the extracapillary space of the cartridge. The pore size of the cartridge is small enough to maintain bacteria in the extracapillary space while allowing the antibiotic to move freely. Media was constantly circulated through the cartridge from a central reservoir. Antibiotic was administered at set concentrations and frequencies shown in Table 1 via NE-1600 syringe pump (New Era) and half-life controlled by media influx and efflux from the central reservoir using Masterflex L/S peristaltic pumps. We defined regimens in Table 1 with continuous or every 12 hours as “short-interval” and with 24 hours, thrice, or once weekly as “extended-interval.” We used an infusion time of 30–60 min, half-life of 3 hours, and specified target C_max_ (μg/mL) of free amikacin as below. We confirmed concentrations by QMS Amikacin assay (Thermo Fisher Scientific) on a Vitros 5600 chemistry analyzer (Ortho Clinical Diagnostics). We first validated the use of this method to quantify amikacin in MH2 broth by testing amikacin in MH2 at known concentrations, 0, 3.75, 15, and 30 µg/mL. Accuracy of measured concentration relative to known concentration was 95%–103% (Table S2). We collected 1 mL of media from the HFIM system just prior to drug infusion (target trough) and at the completion of drug infusion (target C_max_) for amikacin quantification. Measured C_max_ was within 25% of all target values, while trough concentrations were more variable, with accuracy ranging from 93% to 170% (Table S3).

We collected 1 mL of bacteria from the extracapillary space in each cartridge to quantify total CFU/mL on days 1, 2, 3, 5, 7, 10, and 14 post infection by plating serial dilutions onto MH2 agar and resistant colonies on plates containing 24 µg/mL amikacin (3xMIC). During the early time points, samples were plated onto 3xMIC agar, but the concentration of bacteria was below 10^7^ CFU/mL, so baseline-resistant isolates were too infrequent to quantify. Data are reported as ΔCFU_max_, the maximum decrease in CFU compared to control samples obtained at the same time, or day of growth recovery (DGR), the day that CFU had rebounded by ≥1 log_10_ above CFU_min_ in a treatment arm. To more precisely estimate DGR, we linearly interpolated between CFU measured on the surrounding sampling days. We compared growth curves using two-way ANOVA with Tukey’s multiple comparisons test and ΔCFU_max_ and DGR with Student’s *t* test and one-way ANOVA.

### PK-PD modeling, simulation, and PTA

To determine which PK-PD index (%T > MIC, AUC_24_:MIC or Peak:MIC) best predicted ΔCFU_max_ and DGR, we used the minpack.lm package (26) for R 4.2.2 to perform non-linear regression fitted to a sigmoidal model for ΔCFU_max_ and DGR as shown in the equations below.


$$\Delta CFU_{max}=y_{0}-\frac{E_{max}*x^{h}}{EC_{50}^{h}+x^{h}}$$

For $\Delta CFU_{max}$, x is either AUC_24_:MIC, Peak:MIC, or %Time >MIC.


$$DGR=y_{0}+\;\frac{E_{max}*x^{h}}{EC_{50}^{h}+x^{h}}$$

For DGR, *x* is days. For both equations, $y_{0}$ is the value of *y* when *x* is 0; *E_max_* is the maximum effect of *x* on *y*; *EC_50_* is the value of *x* with half-maximal effect on *y*; *h* is the Hill exponent that controls the steepness of the sigmoidal response curve. We calculated AUC_24_ from trapezoidal approximation of amikacin concentrations in the HFIM, peak by extracting the maximal concentration in a dosage interval, and %T > MIC as the duration in one dosage interval that HFIM concentrations exceeded the MIC divided by the dosage interval. We chose the model with the best coefficient of determination (*R*^2^) for predicted vs observed data and lowest AIC, which is a function of the likelihood of the model penalized for overparameterization (27). These are standard criteria for model selection in pharmacometrics (28, 29).

To link exposures in the HFIM to human pediatric dosing, we used our Pmetrics modeling and simulation package for R (30) to simulate from a previously published model of amikacin in children (31). The model had a central and peripheral compartment with clearance from the central compartment. We used the reported mean and CV% for model parameters and a CV% of 100 when not reported. We included weight as in the original model, with a mean (SD) of 40 (10) kg. To avoid negative parameter values, we log transformed the reported linear parameter value distributions according to the following formulae (32): $\log\left(sd\right)=\sqrt{\log\left(\frac{sd^{2}}{mean^{2}}+1\right)}$ and $\log\left(mean\right)=\log\left(\frac{mean}{e^{0.5*{\log\left(sd\right)}^{2}}}\right)$, where *mean* and *sd* are the linear values. We verified our simulated output against Fig. 5 of the original paper (31) and cross-validated the output to be similar to a study of amikacin PK in pediatric patients with CF (33).

For PTA, we used the model to simulate 1,000 concentration-time profiles at daily dosages of 15, 30, 45, 60, and 90 mg/kg (total body weight) per day divided every 8, 12, or 24 hours and TIW. All regimens were simulated for 2 weeks. We chose the target based on the exposure-response analysis above, aiming for a %T > MIC of 40%, which corresponded to the EC_80_ for ΔCFU_max_. For direct comparison with the only prior study on amikacin PK-PD against Mab (14), we also included PTA analysis with a target Peak:MIC of 3.2. For all regimens, we also modeled the probability of amikacin toxicity for both oto- and nephrotoxicity. For ototoxicity, we used the model by Modongo et al. (16) and extrapolated the simulated steady-state daily AUC to a cumulative AUC over 3 and 12 months. For nephrotoxicity, we assessed the probability of a steady-state amikacin trough concentration >10 µg/mL, in accordance with the threshold identified by Yamada et al. (15).

## SUPPLEMENTAL MATERIAL

Supplemental material spectrum.03222-23-s0001.pdf Figure S1 and Tables S1 to S3.

ASM does not own the copyrights to Supplemental Material that may be linked to, or accessed through, an article. The authors have granted ASM a non-exclusive, world-wide license to publish the Supplemental Material files. Please contact the corresponding author directly for reuse.

## References

[1] Lee M-R, Sheng W-H, Hung C-C, Yu C-J, Lee L-N, Hsueh P-R. 2015. Mycobacterium abscessus complex infections in humans. Emerg Infect Dis 21:1638–1646. doi:10.3201/2109.141634 26295364 PMC4550155

[2] Prevots DR, Shaw PA, Strickland D, Jackson LA, Raebel MA, Blosky MA, Montes de Oca R, Shea YR, Seitz AE, Holland SM, Olivier KN. 2010. Nontuberculous mycobacterial lung disease prevalence at four integrated health care delivery systems. Am J Respir Crit Care Med 182:970–976. doi:10.1164/rccm.201002-0310OC 20538958 PMC2970866

[3] Victoria L, Gupta A, Gómez JL, Robledo J. 2021. Mycobacterium abscessus complex: a review of recent developments in an emerging pathogen. Front Cell Infect Microbiol 11:659997. doi:10.3389/fcimb.2021.659997 33981630 PMC8108695

[4] Winthrop KL, Marras TK, Adjemian J, Zhang H, Wang P, Zhang Q. 2020. Incidence and prevalence of nontuberculous mycobacterial lung disease in a large U.S managed care health plan, 2008-2015. Ann Am Thorac Soc 17:178–185. doi:10.1513/AnnalsATS.201804-236OC 31830805 PMC6993793

[5] Cystic Fibrosis Foundation. 2021. Cystic fibrosis foundation patient registry 2020 annual data report. Bethesda, MD

[6] Johansen MD, Herrmann J-L, Kremer L. 2020. Non-tuberculous mycobacteria and the rise of Mycobacterium abscessus. Nat Rev Microbiol 18:392–407. doi:10.1038/s41579-020-0331-1 32086501

[7] Floto RA, Olivier KN, Saiman L, Daley CL, Herrmann J-L, Nick JA, Noone PG, Bilton D, Corris P, Gibson RL, Hempstead SE, Koetz K, Sabadosa KA, Sermet-Gaudelus I, Smyth AR, van Ingen J, Wallace RJ, Winthrop KL, Marshall BC, Haworth CS, US Cystic Fibrosis Foundation and European Cystic Fibrosis Society. 2016. US cystic fibrosis foundation and European cystic fibrosis society consensus recommendations for the management of non-tuberculous mycobacteria in individuals with cystic fibrosis. Thorax 71 Suppl 1:i1–22. doi:10.1136/thoraxjnl-2015-207360 26666259 PMC4717371

[8] Daley CL, Iaccarino JM, Lange C, Cambau E, Wallace RJ, Andrejak C, Böttger EC, Brozek J, Griffith DE, Guglielmetti L, Huitt GA, Knight SL, Leitman P, Marras TK, Olivier KN, Santin M, Stout JE, Tortoli E, van Ingen J, Wagner D, Winthrop KL. 2020. Treatment of nontuberculous mycobacterial pulmonary disease: an official ATS/ERS/ESCMID/IDSA clinical practice guideline. Clin Infect Dis 71:905–913. doi:10.1093/cid/ciaa1125 32797222 PMC7768745

[9] Chen J, Zhao L, Mao Y, Ye M, Guo Q, Zhang Y, Xu L, Zhang Z, Li B, Chu H. 2019. Clinical efficacy and adverse effects of antibiotics used to treat Mycobacterium abscessus pulmonary disease. Front. Microbiol 10. doi:10.3389/fmicb.2019.01977 PMC6716072 31507579

[10] Kwak N, Dalcolmo MP, Daley CL, Eather G, Gayoso R, Hasegawa N, Jhun BW, Koh W-J, Namkoong H, Park J, Thomson R, Ingen J, Zweijpfenning SMH, Yim J-J. 2019. Mycobacterium abscessus pulmonary disease: individual patient data meta-analysis. Eur Respir J 54. doi:10.1183/13993003.01991-2018 30880280

[11] Scaglione F, Paraboni L. 2006. Influence of pharmacokinetics/pharmacodynamics of antibacterials in their dosing regimen selection. Expert Rev Anti Infect Ther 4:479–490. doi:10.1586/14787210.4.3.479 16771624

[12] Turnidge J. 2003. Pharmacodynamics and dosing of aminoglycosides. Infect Dis Clin North Am 17:503–528, doi:10.1016/s0891-5520(03)00057-6 14711074

[13] Eyler RF, Shvets K. 2019. Clinical pharmacology of antibiotics. CJASN 14:1080–1090. doi:10.2215/CJN.08140718 30862698 PMC6625637

[14] Ferro BE, Srivastava S, Deshpande D, Sherman CM, Pasipanodya JG, van Soolingen D, Mouton JW, van Ingen J, Gumbo T. 2016. Amikacin pharmacokinetics/pharmacodynamics in a novel hollow-fiber Mycobacterium abscessus disease model. Antimicrob Agents Chemother 60:1242–1248. doi:10.1128/AAC.02282-15 PMC4775936 26643339

[15] Yamada T, Fujii S, Shigemi A, Takesue Y. 2021. A meta-analysis of the target trough concentration of gentamicin and amikacin for reducing the risk of nephrotoxicity. J Infect Chemother 27:256–261. doi:10.1016/j.jiac.2020.09.033 33077364

[16] Modongo C, Pasipanodya JG, Zetola NM, Williams SM, Sirugo G, Gumbo T. 2015. Amikacin concentrations predictive of ototoxicity in multidrug-resistant tuberculosis patients. Antimicrob Agents Chemother 59:6337–6343. doi:10.1128/AAC.01050-15 26248372 PMC4576092

[17] Bhatt J, Jahnke N, Smyth AR. 2019. Once-daily versus multiple-daily dosing with intravenous aminoglycosides for cystic fibrosis. Cochrane Database Syst Rev 9:CD002009. doi:10.1002/14651858.CD002009.pub7 31483853 PMC6726357

[18] Moore RD, Lietman PS, Smith CR. 1987. Clinical response to aminoglycoside therapy: importance of the ratio of peak concentration to minimal inhibitory concentration. J Infect Dis 155:93–99. doi:10.1093/infdis/155.1.93 3540140

[19] Kato H, Hagihara M, Hirai J, Sakanashi D, Suematsu H, Nishiyama N, Koizumi Y, Yamagishi Y, Matsuura K, Mikamo H. 2017. Evaluation of amikacin pharmacokinetics and pharmacodynamics for optimal initial dosing regimen. Drugs R D 17:177–187. doi:10.1007/s40268-016-0165-5 28063020 PMC5318333

[20] Craig WA. 2011. Optimizing aminoglycoside use. Crit Care Clin 27:107–121. doi:10.1016/j.ccc.2010.11.006 21144989

[21] Clinical and Laboratory Standards Institute. 2011. M24-A2 susceptibility testing of mycobacteria, nocardiae, and other aerobic actinomycetes; approved standard. 2nd ed 31339680

[22] Drusano GL, Myrick J, Maynard M, Nole J, Duncanson B, Brown D, Schmidt S, Neely M, Scanga CA, Peloquin C, Louie A. 2018. Linezolid kills acid-phase and nonreplicative-persister-phase Mycobacterium tuberculosis in a hollow-fiber infection model. Antimicrob Agents Chemother 62:e00221-18. doi:10.1128/AAC.00221-18 29866864 PMC6105790

[23] Drusano GL, Rogers S, Brown D, Peloquin CA, Neely M, Yamada W, Kim S, Almoslem M, Schmidt S, Louie A. 2021. Dose fractionation of moxifloxacin for treatment of tuberculosis: impact of dosing interval and elimination half-life on microbial kill and resistance suppression. Antimicrob Agents Chemother 65. doi:10.1128/AAC.02533-20 PMC8097450 33468465

[24] Drusano GL, Sgambati N, Eichas A, Brown DL, Kulawy R, Louie A, Low DE. 2010. The combination of rifampin plus moxifloxacin is synergistic for suppression of resistance but antagonistic for cell kill of Mycobacterium tuberculosis as determined in a hollow-fiber infection model. mBio 1:e00139-10. doi:10.1128/mBio.00139-10 20802826 PMC2925073

[25] Nicasio AM, Bulitta JB, Lodise TP, D’Hondt RE, Kulawy R, Louie A, Drusano GL. 2012. Evaluation of once-daily vancomycin against methicillin-resistant Staphylococcus aureus in a hollow-fiber infection model. Antimicrob Agents Chemother 56:682–686. doi:10.1128/AAC.05664-11 22083484 PMC3264248

[26] Elzhov TV, Mullen KM, Spiess A-N, Bolker B. 2022. minpack.lm: R interface to the levenberg-marquardt nonlinear least-squares algorithm found in MINPACK, plus support for bounds

[27] Akaike H. 1974. A new look at the statistical model identification. IEEE Trans Automat Contr 19:716–723. doi:10.1109/TAC.1974.1100705

[28] Mould DR, Upton RN. 2013. Basic concepts in population modeling, simulation, and model-based drug development-part 2: introduction to pharmacokinetic modeling methods. CPT Pharmacometrics Syst Pharmacol 2:e38. doi:10.1038/psp.2013.14 23887688 PMC3636497

[29] Goutelle S, Woillard J-B, Neely M, Yamada W, Bourguignon L. 2022. Nonparametric methods in population pharmacokinetics. J Clin Pharmacol 62:142–157. doi:10.1002/jcph.1650 33103785

[30] Neely MN, van Guilder MG, Yamada WM, Schumitzky A, Jelliffe RW. 2012. Accurate detection of outliers and subpopulations with pmetrics, a nonparametric and parametric pharmacometric modeling and simulation package for R. Ther Drug Monit 34:467–476. doi:10.1097/FTD.0b013e31825c4ba6 22722776 PMC3394880

[31] Alhadab AA, Ahmed MA, Brundage RC. 2018. Amikacin pharmacokinetic-pharmacodynamic analysis in pediatric cancer patients. Antimicrob Agents Chemother 62:12. doi:10.1128/AAC.01781-17 PMC5913936 29358293

[32] Feng C, Wang H, Lu N, Chen T, He H, Lu Y, Tu XM. 2014. Log-transformation and its implications for data analysis. Shanghai Arch Psychiatry 26:105–109. doi:10.3969/j.issn.1002-0829.2014.02.009 25092958 PMC4120293

[33] Caceres Guido P, Perez M, Halac A, Ferrari M, Ibarra M, Licciardone N, Castaños C, Gravina LP, Jimenez C, Garcia Bournissen F, Schaiquevich P. 2019. Population pharmacokinetics of amikacin in patients with pediatric cystic fibrosis. Pediatr Pulmonol 54:1801–1810. doi:10.1002/ppul.24468 31402602

