# Supplementary material for: Time-dependent pharmacodynamics of amikacin on Mycobacterium abscessus growth and resistance emergence
Source: Microbiol Spectr. 2024 Jan 18;12(2):e03222-23. doi: 10.1128/spectrum.03222-23 (PMC10846206; doi:10.1128/spectrum.03222-23)
Supplement: Supplemental material — Figure S1 and Tables S1 to S3. [file spectrum.03222-23-s0001.pdf]

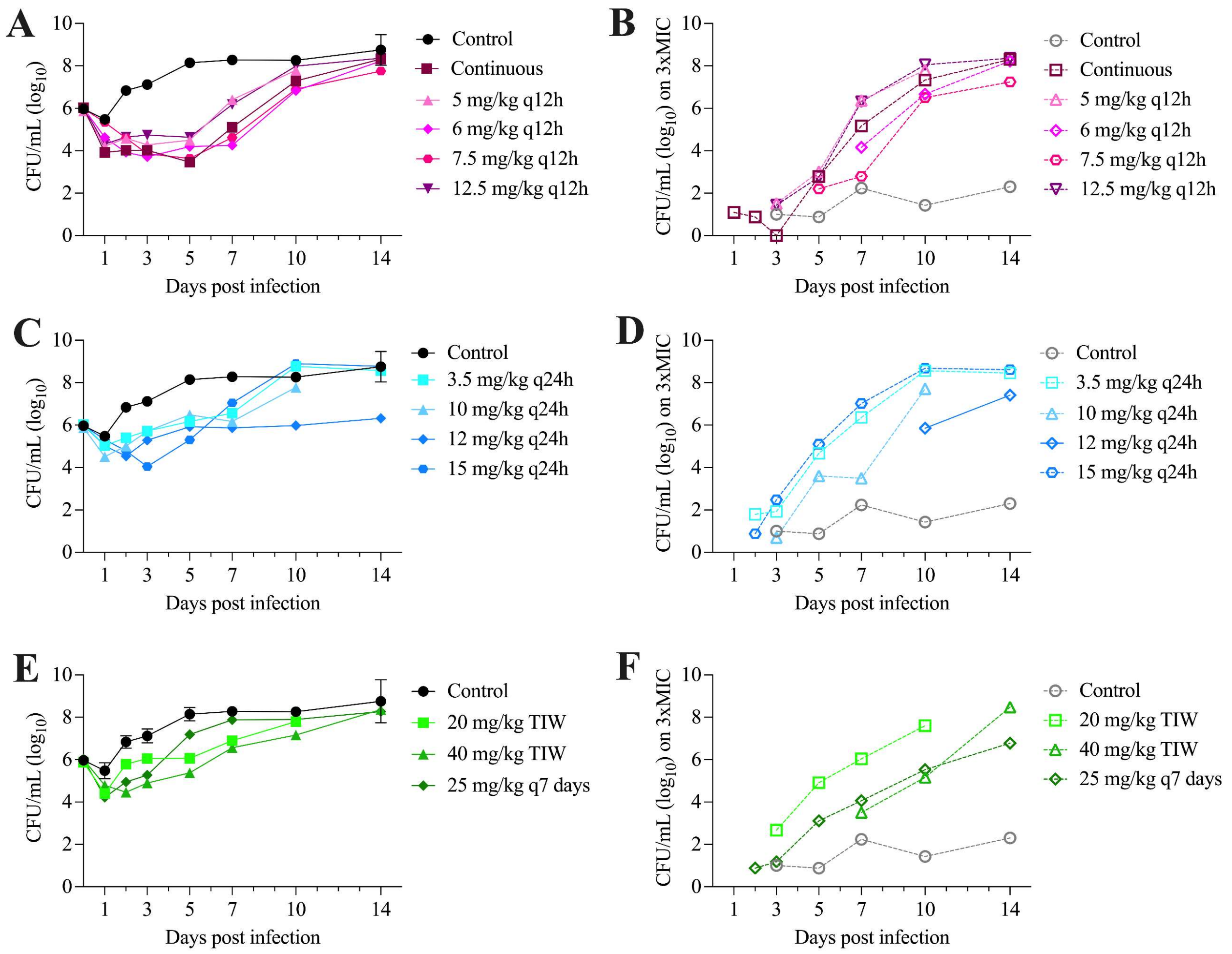

Figure S1. Growth of Mab in the HFIM with amikacin treatment. Colony forming units (CFU) on MH2 agar plates without (closed symbols, **A, C, E**) or with 3xMIC amikacin (open symbols/dashed lines, **B, D, F**) over 14 days. Amikacin was administered at different concentrations and dosing frequencies to approximate human pediatric doses with an infusion time of 30-60 minutes and half-life of 3 hours. Control arms did not receive antibiotic or vehicle. Amikacin dosing frequencies were grouped into <24 hours (pink; continuous infusion, n=1, or every 12 hours, q12h, n=4; **A, B**), every 24 hours (blue; q24h, n=4, **C, D**), or >24 hours (green; thrice weekly, TIW, n=2; or every 7 days, q7 days, n=1; **E, F**). During the early time points, resistant isolates were below the limit of detection in the quantity of bacteria plated on 3xMIC amikacin plates. Data shown in **B**, **D**, and **F** are only reported for resistant isolates that were above the threshold to allow quantification.

Table S1. Akaike information criterion scores for PK-PD indices corresponding to microbial kill

PK-PD parameter	ΔCFU_{max}	Day 1	Day 2	Day 3	Day 5	Day 7	Day 10	Day 14
%T>MIC	24.2	22	20.7	29.7	33.4	36.7	35.6	33.3
AUC:MIC	36.3	NC	24.4	NC	40.6	NC	NC	NC
Peak:MIC	NC	NC	NC	NC	NC	NC	NC	NC

Notes: Values shown are the Akaike information criterion (AIC) score at each timepoint based on non-linear regression analysis of delta CFU with an inhibitory Emax model. Lower AIC represent the more likely model. NC = no convergence of the model and unable to calculate AIC.

Table S2. Validation of amikacin assay for amikacin sulfate in MH2 broth

Standard (μg/mL)*	Tube 1	Tube 2	Accuracy (%)
0	<1.35	<1.35	-
3.75	3.85	3.89	**103.20**
15	15.75	15.17	**103.07**
30	27.59	29.31	**94.83**

*Concentration of free amikacin from amikacin sulfate diluted in MH2

Table S3. Measured free amikacin concentrations on day 7 for each dosing regimen

**Frequency**	**Equivalent Daily Dose**	**Target Cmax (μg/mL)**	**Measured Cmax (μg/mL)**	**Accuracy (%)**	**Target trough (μg/mL)**	**Measured Trough (μg/mL)**	**Accuracy (%)**
No amikacin	Untreated	0	<1.35	-	0	<1.35	-
Continuous	20 mg/kg	15	13.48	89.9	15	13.90	92.7
q12h	10 mg/kg	20.3	21.3	104.9	1.4	1.9	135.7
q12h	12 mg/kg	22.3	20.0	89.6	1.6	2.2	137.5
q12h	15 mg/kg	23.3	17.5	75.1	1.8	1.9	104.4
q12h	25 mg/kg	50.5	40.51	80.2	3.6	6.09	169.2
q24h	3.5 mg/kg	12.6	9.54	75.7	0.1	<1.35	-
q24h	10 mg/kg	40.1	39.5	98.5	0	<1.35	-
q24h	12 mg/kg	45.7	42.0	91.9	0	<1.35	-
q24h	15 mg/kg	52.5	40.9	77.9	0	<1.35	-
TIW	20 mg/kg	71.2	69.2	97.2	0	<1.35	-
TIW	40 mg/kg	149.6	138.0	92.2	0	<1.35	-
q7 days	25 mg/kg	90	77.28	85.9	0	<1.35	-
